# lncRNA FEZF1‐AS1 regulates biological behaviors of cervical cancer by targeting miRNA‐1254

**DOI:** 10.1002/fsn3.2315

**Published:** 2021-07-14

**Authors:** Miao Liang, Yongkang Li, Tingting Dai, Cheng Chen

**Affiliations:** ^1^ Department of gynaecology and obstetrics Chongqing General Hospital University of Chinese Academy of Sciences Chongqing China

**Keywords:** cervical cancer, lncRNA FEZE1‐AS1, miR‐1254, Smurf 1

## Abstract

**Aim:**

The purpose of this research was to evaluate lncRNA FEZF1‐AS1 in cervical cancer development and clinical significance.

**Materials and methods:**

Collecting cervical cancer tissues, measuring FEZF1‐AS1 expression, and analysis correlation between FEZF1‐AS1 and prognosis. In cell vitro study, using MTT assay to measure cell proliferation, evaluating cell apoptosis by flow cytometry, measuring cell invasion and migration by Transwell and wound healing assay; lncRNA FEZF1‐AS1 and miR‐1254 gene expressions were evaluated by RT‐qPCR assay; relative protein (Smurf1, E‐cadherin, Vimentin, N‐cadherin, AKT, p‐AKT, c‐Myc, and ZEB1) expressions were measured by Western blot assay. The correlation among FEZF1‐AS1, miR‐1254, and Smurf1 were analysis by dual luciferase reporter gene assay.

**Results:**

By clinical analysis, lncRNA FEZF1‐AS1 was high expression in cervical cancer tissues and high expression was closely correlated with poor prognosis in cervical cancer patients. In vitro study, the SiHa and HeLa cell biologically including cell proliferation, migration, and invasion of si‐FEZF1‐AS1 group which knockdown lncRNA FEZF1‐AS1 were significantly depressed (*p* < .001, respectively). However, with miR‐1254 expression inhibiting, the cell biological activities were significantly increased in si‐FEZF1‐AS1+miRNA inhibitor groups (*p* < .001, respectively).

**Conclusion:**

lncRNA FEZF1‐AS1 might be an oncological role in cervical cancer; lncRNA FEZF1‐AS1 knockdown had antitumor effects with miR‐1254 activating in cervical cancer by in vitro study.

## Graphical abstract

lncRNA FEZF1‐AS1 might be an oncological role in cervical cancer, lncRNA FEZF1‐AS1 knockdown had anti‐tumor effects with miR‐1254 activating in cervical cancer by vitro study.

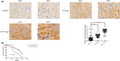

## 1 INTRODUCTION

Cervical cancer (CC) is a leading cause of death in women around the world. In Asia, CC is the second deadliest malignant tumor in women following breast cancer (Vaccarella et al., 2017). Approximately a total of 527,600 new cases of CC were found in 2012; and 265,700 people died from CC in the same year (Siegel et al., 2019). The prevalence is higher in women in low‐ and middle‐income countries (LMIC) (Siegel et al., 2019); (Ginsburg et al., 2017). The persistent high‐risk infection of human papillomavirus (HPV) is a major pathogenic factor for CC. More than 95% patients with CC are HPV‐positive (Walboomers et al., 1999). The pathogenesis of CC, from HPV infection, cervical intraepithelial neoplasia (CIN), and distant metastasis from carcinoma in situ to invasive carcinoma, is complex and accompanied by multistep and polygenic regulatory abnormalities. Besides, this process often involves disorders of the expression of multiple coding and noncoding genes (Chen and Junying, 2018; Qian et al., 2017; Shang et al., 2019). Although comprehensive therapies have improved the clinical therapeutic effect of CC, the prognosis of advanced cervical cancer (ACC) still needs further enhancement. Therefore, it is necessary to explore the functional molecules and therapeutic targets associated with CC (Origoni et al., 2015). In 2002, Okazaki et al. (Okazaki et al., 2002) were the first to discover a novel transcript type (i.e., long noncoding RNA, lncRNA) in a large‐scale sequencing of the mouse full‐length complementary DNA (cDNA) library. lncRNAs are a type of functional RNA molecule over 200 nt in length and almost lose the ability to encode proteins due to the lack of open reading frames (ORF) (Mattick and Rinn, 2015; Ponting et al., 2009). However, lncRNAs can regulate gene expression at different levels in the form of RNA (Bartonicek et al., 2016):

lncRNAs mediate protein–protein interactions (PPIs) as a “scaffold” (Mattick and Rinn, 2015; Morris and Mattick, 2014), promote the binding of proteins to gene promoters as a “guide,” regulate the transcription of neighboring genes as a “signal,” and competitively bind to miRNAs or proteins as a “bait” to influence the transcription of mRNAs (Salmena et al., 2011). Therefore, lncRNAs play a key role in cell growth, development, aging, and death (Mercer et al., 2009). Moreover, more and more evidence showed that lncRNAs are involved in the tumorigenesis and development of CC: lncRNA‐CTS regulates ZEB2 through a competitive endogenous RNA mechanism, affecting the epithelial–mesenchymal transition (EMT) of CC cells (Feng et al., 2019); lncRNA‐CCDST promotes the interaction between MDM2 and DHX9 as a “scaffold,” inducing angiogenesis and invasive metastasis of CC (Ding et al., 2019). A new study showed that lncRNA FEZ family zinc finger 1 antisense RNA1 (FeZF1‐AS1) is a key regulator of human cancers (Zhou et al., 2019; Wang et al., 2018). However, the expression and relevant mechanisms of FEZB1‐AS1 in CC are still unclear.

miR‐1254 is a cancer‐related gene. Some studies have shown that the expression of miR‐1254 in gastric cancer is down‐regulated; the overexpression of miR‐1254 can significantly inhibit the proliferation, metastasis, and invasion of gastric cancer cells (Jiang et al., 2019). LncRNA DCST1 antisense RNA1 (DCST1‐AS1) promotes the proliferation and growth of tumor cells by adsorbing miR‐1254 and regulating the expression of FAIM2 (Chen et al., 2019). Bioinformatics predictions showed that miR‐1254 is a potential target gene of FEZF1‐AS1, but it is still unclear whether FEZF1‐AS1 affects the biological behaviors of CC cells by regulating the expression of miR‐1254. The study is aimed at exploring the biological functions and regulatory mechanism of FEZF1‐AS1 in CC by analyzing the expression of lncRNA FEZF1‐AS1 in CC and its correlations with the prognosis of patients with CC and then by interfering with the expression of FEZF1‐AS1 or overexpressing miR‐1254 in CC cells.

## 2 DATA AND METHODS

### 2.1 Tissue specimens

The study was approved by the Ethics Committee of the hospital. The pathological tissues of a total of 125 patients treated in Chongqing General Hospital from February 2012 to February 2017 were selected. These patients were informed of the study and signed informed consent forms. The patients enrolled were diagnosed with CC and received radical resection in our hospital. The clinical staging was based on the 2009 FIGO clinical staging criteria for CC. All tumor tissues and adjacent normal tissues (ANTs) were directly sampled from patients. ANTs were more than 5cm from tumor edges; and significantly calcified or necrotic parts were avoided; after the specimens were sampled, they were placed in freezing tubes and immediately stored in liquid nitrogen for subsequent experiments. These patients did not receive preoperative chemoradiotherapy and had no history of other tumors or major organ diseases. Patients were followed up by phone (5 patients were not followed up) to record their prognostic survival data and overall survival (OS): TNM staging, stages I‐II: 73 cases and stages III‐IV: 47 cases. Patients were followed up since half a year after the operation. The endpoint of follow‐up was death of patients or May 31, 2020, the deadline of this study.

### 2.2 Reagents and materials

The RPMI‐1640 medium and DMEM were purchased from Gibco (USA); Lipofectamine 2000 was purchased from Invitrogen (USA); the MTT kit was purchased from Sigma (USA); the cell death detection kit was purchased from Dalian Meilun Biotechnology Co., Ltd.; the reverse transcription kit and SYBR Green qPCR Master Mix were purchased from Takara (Dalian); Transwell chambers and Matrigel were purchased from BD (USA); the dual luciferase reporter gene assay kit, TRIzol reagent, and BCA kit were purchased from Shanghai Beyotime Biotechnology Co., Ltd.; the FEZF1‐AS1 small interfering RNA (si‐FEZF1‐AS1), small interfering RNA controls (si‐NC), miR‐1254 mimics, mimic controls (miR‐con) and miRNA inhibitor empty vector plasmid (pcDNA), FEZF1‐AS1 overexpression vector (pcDNA‐FEZF1‐AS1), wild‐type luciferase reporter gene vector (WT‐FEZF1‐AS1), and mutant luciferase reporter gene vector (MUT‐FEZF1‐AS1) were provided by Sangon Biotech (Shanghai) Co., Ltd.; Smurf1, E‐cadherin, Vimentin, N‐cadherin, AKT, p‐AKT, c‐Myc, ZEB1, and GAPDH antibodies and secondary antibodies were purchased from Abcam (USA).

### 2.3 In situ hybridization (ISH)

In order to detect the expression of lncRNA FEZF‐1‐AS1 in CC tissues and ANTs, ISH was performed according to the kit instructions (the kit was purchased from Boster, Wuhan). The digoxin‐labeled lncRNA FEZF1‐AS1 probe (1:400) was added to paraffin sections of tissues, after which tissues were incubated at 55℃ for 1h and then washed. Then, the tissues were sealed with a reagent for 1h; after removing the reagent, tissue sections were placed in TBST containing anti‐digoxin antibodies (1:200) and incubated at 37℃ for 1h. Finally, HE staining was performed; and the dyeing effects were observed and photographed under the microscope. The image analysis software ImageJ was used to analyze IOD values in tissues.

### 2.4 Cell strains and cell culture

CC cell strains (SiHa and HeLa) were purchased from ATCC (USA). 1% double antibodies to penicillin–streptomycin and 10% fetal bovine serum (FBS) were added to the DMEM. The cells were routinely placed in an incubator containing 5% CO_2_ at 37℃ and experienced a passage every 2~3d, until the cells reached the logarithmic phase.

### 2.5 Cell transfection and experimental grouping

si‐FEZF1‐AS1, si‐NC, and miR‐1254 were transfected into Siha or Hela cells by lipofection. The transfected cells were labeled as Si‐FezF1‐AS1, Si‐NC, and Mir‐1254. 48h after the transfection, the transfection effect was detected by qRT‐PCR. Follow‐up studies were performed. The normally cultured CC cells were used as a NC group. In order to further verify whether lncRNA FEZF1‐AS1 affects the biological behaviors of CC cells by regulating the expression of miR‐1254, si‐FEZF1‐AS1, and miRNA‐1254, inhibitors were cotransfected into CC cell strains. The transfected cells were labeled as NC, si‐FEZF1‐AS1, miR‐1254, and si‐FEZF1‐AS1+miRNA inhibitor.

### 2.6 Detection of the expression of FEZF1‐AS1 and miR‐1254 by RT‐qPCR

The transfected CC cells in each group were collected. The reagent TRIzol was used to extract the total RNA, which was reversely transcribed to cDNA. RT‐qPCR was performed. FEZF1‐AS1 primer: upstream: 5’‐AGA GGC TAT GAC TCA GGG TT‐3’, downstream: 5’‐TGT TGC TCC ACA GTA AAG GT‐3’; reference β‐actin primer: upstream: 5’‐GCA TCG TCA CCA ACT GGG AC‐3’, downstream: 5’‐ACC TGG CCG TCA GGC AGC TC‐3’; miR‐1254 primer: upstream: 5’‐AGC CTG GAA GCT GGA GCC TGC AGT‐3’, downstream: 5’‐GCG AGC ACA GAA TTA ATA CGA C‐3’; reference U6 primer: upstream: 5’‐CTC GCT TCG GCA GCA CA‐3’, downstream: 5’‐AAC GCT TCA CGA ATT TGC GT‐3’. All primers were synthesized by Sangon Biotech (Shanghai) Co., Ltd. The reaction conditions for the PCR were 95℃ and 3min, 95℃ and 30s, 58℃ and 30s, and 72℃ and 30s for a total of 35 cycles. The relative expression quantity of the target genes was calculated by 2 ^‐ ΔΔCt^.

### 2.7 Detection of cell proliferation rates by MTT testing

The CC cells from all groups were inoculated into 96‐well plate according to 2 × 10^3^/well. 48h after the transfection, 20 μL MTT solution (5 mg/ml) was added to each well. Cells were incubated in an incubator for 4h. The supernatant was discarded. Then, 150 μL DMSO was added to each well, after which cells were further incubated for 2h. An automatic microplate reader was used to determine the absorbance (A) at the wavelength of 490nm (zero setting was performed in a blank well).

### 2.8 Flow cytometry (FCM)

48h after the corresponding treatment of cells in each group, after trypsin digestion, cells in each group were collected by centrifugation and washed with PBS. Binding buffer was added. Cells were resuspended to make the cell concentration reach 1*106 /ml. 100μL cell suspension was taken and added to a flow tube. 5 μL Annexin VFITC and 5 μL PI were added. The mixture was mixed well and incubated at a room temperature and away from light for 15min. 400μL binding buffer was added to each tube. After mixing, cells were placed on the flow cytometer to detect the apoptosis.

### 2.9 Transwell assay

Chambers coated with Matrigel were placed in a 24‐well plate. 300μL preheated serum‐free medium was added to the upper chamber. Chambers were allowed to stand at ambient temperature for 15min. When the Matrigel was hydrated, the remaining culture solution was removed. 48h after the transfection, cells were digested, washed with PBS, and resuspended with serum‐free medium to adjust the cell density to 3*105 /ml. 200μL cell suspension was taken and added to the Transwell chamber. 500μL medium containing 10% FBS was added to the lower chamber of the 24‐well plate. The mixture was routinely cultured for 12h. The cells in the upper chamber were wiped off with a cotton swab and stained with 0.1% crystal violet. The Transwell chamber was inverted under a microscope for observation and photography. Five fields were randomly selected to count invading cells. The mean was calculated.

### 2.10 Would healing assay

When cells in all groups were treated accordingly for 48h, 3*10^4^ cells were added to each hole. The next day, low‐concentration serum‐containing medium was used. A scratch tester was used to push the scratch upward from the central part at the lower end of the 6‐well plate. Cells were rinsed with the serum‐free medium twice, after which the low‐concentration serum‐containing medium was added for photography for 0h. Cells were placed in an incubator with 5% CO_2_ at 37℃ for incubation and photographed at 24h and 48h. The wound healing rate of cells in each group was calculated.

### 2.11 Western blot (WB) assay

The total protein of cells in each group was extracted. The BCA was used to quantify protein concentration. 30Μg protein was taken for SDS‐PAGE electrophoresis and then transferred to PVDF membrane. The corresponding antibodies were added. The protein was incubated at 4℃ overnight. Secondary antibodies (diluted concentration 1:1,000) were added. ECL development, exposure, and photography were performed. The gel imaging analysis system and the software ImageJ were used to analyze the gray value of each strip.

### 2.12 Dual luciferase reporter gene assay

The FEZF1‐AS1‐3’‐UTR‐containing WT‐FEZF1‐AS1 and MUT‐FEZF1‐AS1, or the Smurf1‐3’‐UTR‐containing WT‐Smurf1 and MUT‐Smurf1, were cotransfected into CC cells, respectively, with miR‐NC and miR‐1254 by lipofection. 48h later, the luciferase activity of CC cells was determined by the dual luciferase reporter gene assay.

### 2.13 Statistical analysis

The statistical software SPSS 23.0 was used for statistical analysis. The measurement data were expressed as mean±*SD*. The paired *t* test was applied to analyze the differences in the expression of lncRNA FEZF1‐AS1 between tumor tissues and ANTs. One‐way ANOVA was used for data comparison among groups. The chi‐square test was used to analyze the counting data. The Kaplan–Meier method was taken to draw a survival curve. One‐way analysis of variance was used for comparison among groups. The SNK‐q test was applied for further comparison among groups. The difference was statistically significant for *p* <.05.

## 3 RESULTS

### 3.1 Analysis of FEZF1‐AS1 expression and correlation with prognosis

**FIGURE 1:**
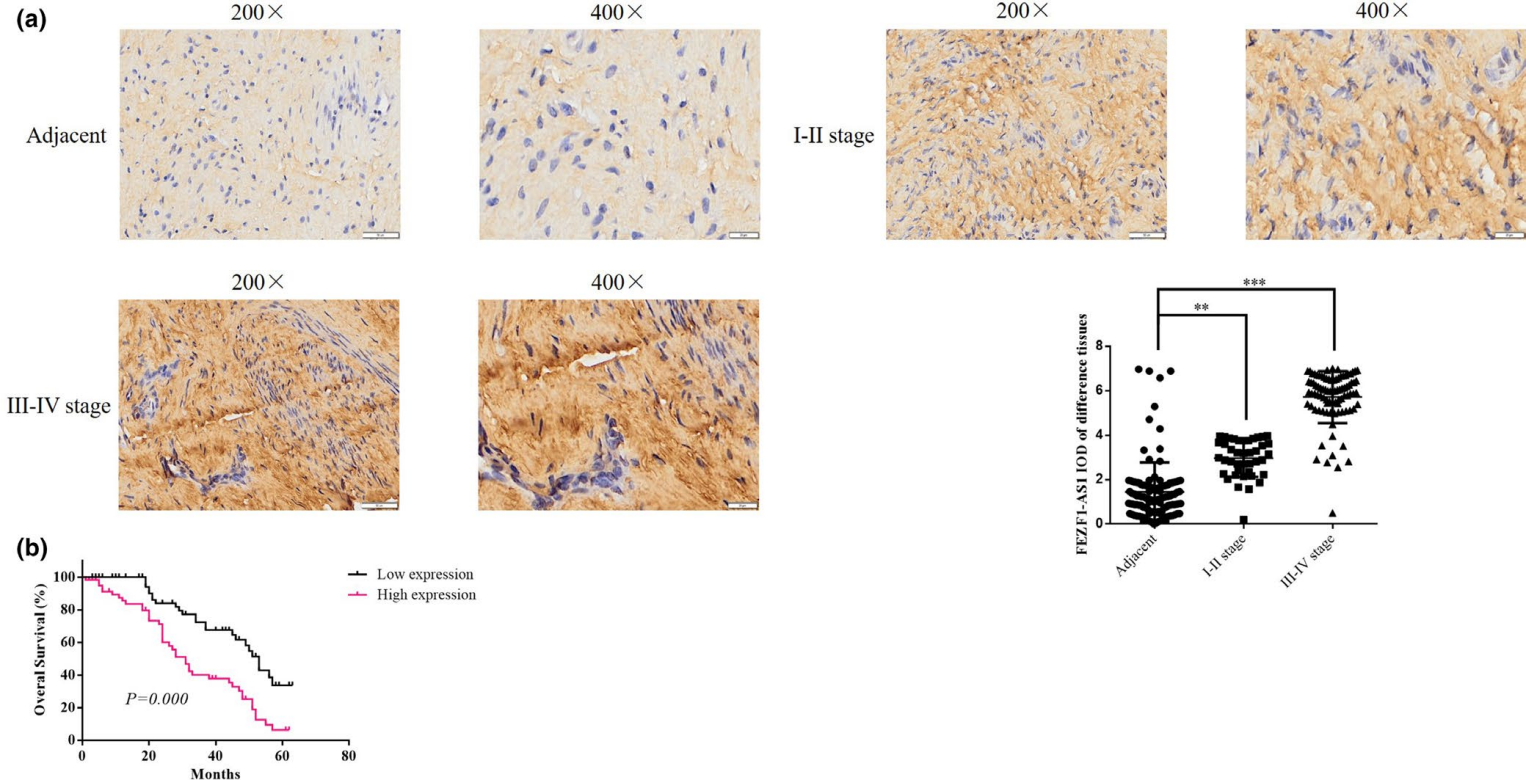
FEZF1‐AS1 expression and correlation with prognosis. (a) FEZF1‐AS1 expression in tissues by ISH assay. **p <.01, ***p <.001, compared with adjacent. (b) Correlation between FEZF1‐AS1 and prognosis

The results of ISH detection showed that the expression of FEZF1‐AS1 in CC tissues at stages I‐II and stages III‐IV was significantly higher than that in ANTs (*p* <.01, respectively, Figure 1a). After RT‐qPCR detection of the 120 cancer tissues included in this study, 120 patients were divided into a low expression group and a high expression group according to the median. The results of analysis by the Kaplan–Meier method indicated that the OS in the low expression group was significantly longer than that in the high expression group (*p* <.000, Figure 1b). One‐way ANOVA was used to analyze the correlations between changes in the expression of lncRNA FEZF1‐AS1 and clinicopathologic features of CC. The data showed that the expression of lncRNA FEZF1‐AS1 was closely related to the histological origin, tumor differentiation degree, and TNM stages of CC (*p* <.05, respectively, Table 1). The expression of lncRNA FEZF1‐AS1 was irrelevant to other clinicopathologic features (age, HPV, or tumor size) (*p* >.05, Table 1).

**TABLE 1:** lncRNA FEZF1‐AS1 expression and clinicopathological features (n,%)

	lncRNA FEZF1‐AS1		*χ^2^*	*P* value
	High expression (*n* = 62)	Low expression (*n* = 58)		
Year old			0.311	0.596
≥45 year old	30 (48.4%)	31 (53.4%)		
<45 year old	32 (51.6%)	27 (46.6%)		
Histological origin			12.863	0.000
Adenocarcinoma	7 (11.3%)	23 (39.7%)		
Squamous carcinoma	55 (88.7%)	35 (60.3%)		
Tumor size			0.933	0.364
≥4 cm	30 (48.4%)	23 (39.7%)		
<4 cm	32 (51.6%)	35 (60.3%)		
HPV infection			0.404	0.719
Negative	5 (8.1%)	3 (5.2%)		
Positive	57 (91.9%)	55 (94.8%)		
Tumor differentiation degree			10.048	0.000
Middle and High	26 (41.9%)	41 (70.7%)		
Low	36 (58.1%)	17 (29.3%)		
Lymph node metastasis			1.357	0.168
Yes	25 (41.3%)	26 (29.5%)		
No	37 (58.7%)	62 (70.5%)		
TNM stage			5.589	0.029
I‐II stage	25 (40.3%)	16 (27.6%)		
III‐IV stage	37 (59.7%)	42 (72.4%)		

### 3.2 Influence of the knockout of FEZF1‐AS1 on proliferation of CC cells

**FIGURE 2:**
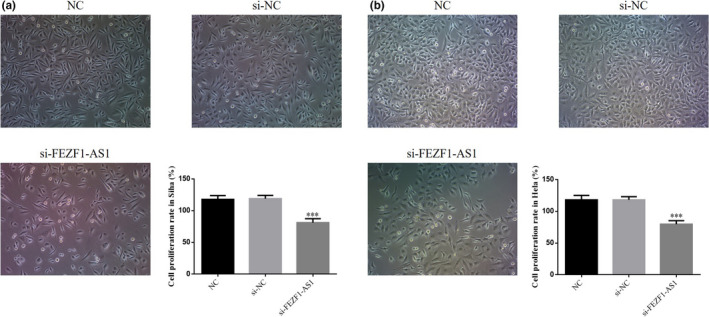
FEZF1‐AS1 knockdown on proliferation of CC cells. (a) Cell proliferation rate in Siha. (b) Cell proliferation rate in Hela. ***p <.001, compared with NC

The results of MTT testing showed that the proliferation rates of SiHa and HeLa cells in the FEZF1‐AS1‐knocked out group were significantly lower than those in the NC group (*p* <.001, respectively, Figure 2a&b), while no significant difference in the proliferation rate was found between Siha cells and Hela cells in the si‐NC group (*p* >.05), indicating that the transfection process did not impair cell proliferation.

### 3.3 Influence of the knockout of FEZF1‐AS1 on apoptosis of CC cells

**FIGURE 3:**
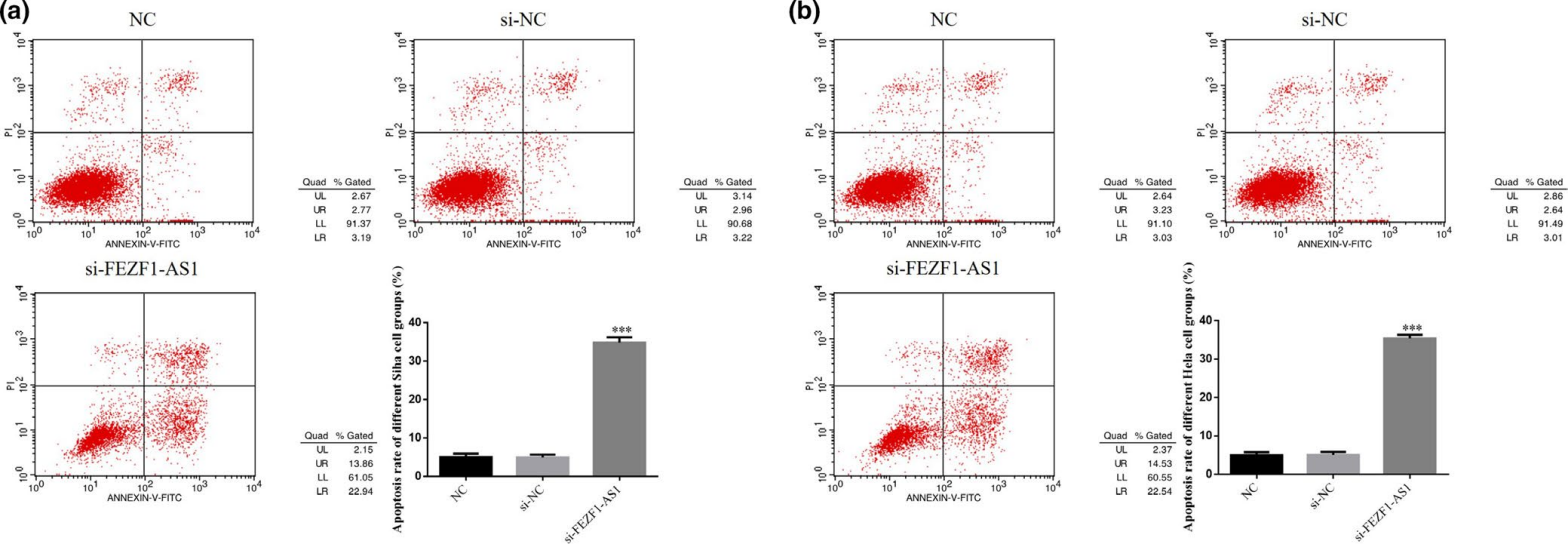
Knockout of FEZF1‐AS1 on apoptosis of CC cells. (a) Apoptosis cell rate in Siha. (b) Apoptosis cell rate in Hela. ***p <.001, compared with NC

The results of the FCM showed that the apoptosis rates of SiHa and HeLa cells in the FEZF1‐AS1‐knocked out group were significantly higher than those in the NC group (*p* <.001, respectively, Figure 3a&b), while no significant difference in the apoptosis rate was found between Siha cells and Hela cells in the si‐NC group (*p* >.05), indicating that the transfection process did not affect cell apoptosis.

### 3.4 Influence of the knockout of FEZF1‐AS1 on invasion of CC cells

**FIGURE 4:**
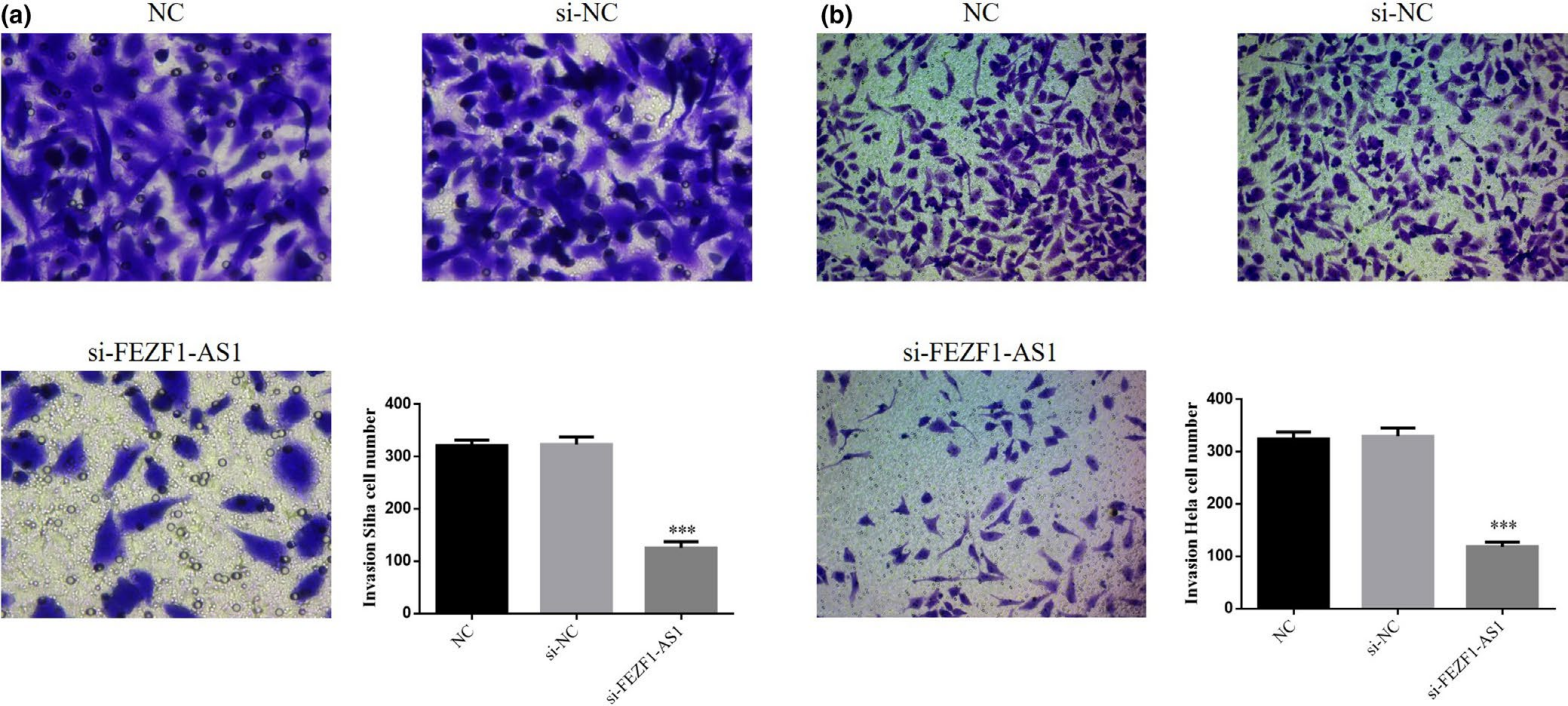
Knockout of FEZF1‐AS1 on cell invasion. (a) Invasion Siha cell number. (b) Invasion Hela cell number. ***p <.001, compared with NC

According to the Transwell results, the invasion of SiHa and HeLa cells in the FEZF1‐AS1‐knocked out group was significantly lower than that in the NC group (*p* <.001, respectively, Figure 4a&b), while SiHa and HeLa cells in the si‐NC group had no significant difference in invasion (*p* >.05), indicating that the transfection process did not affect the invasion of SiHa and HeLa cells.

### 3.5 Influence of the knockout of FEZF1‐AS1 on migration of CC cells

**FIGURE 5:**
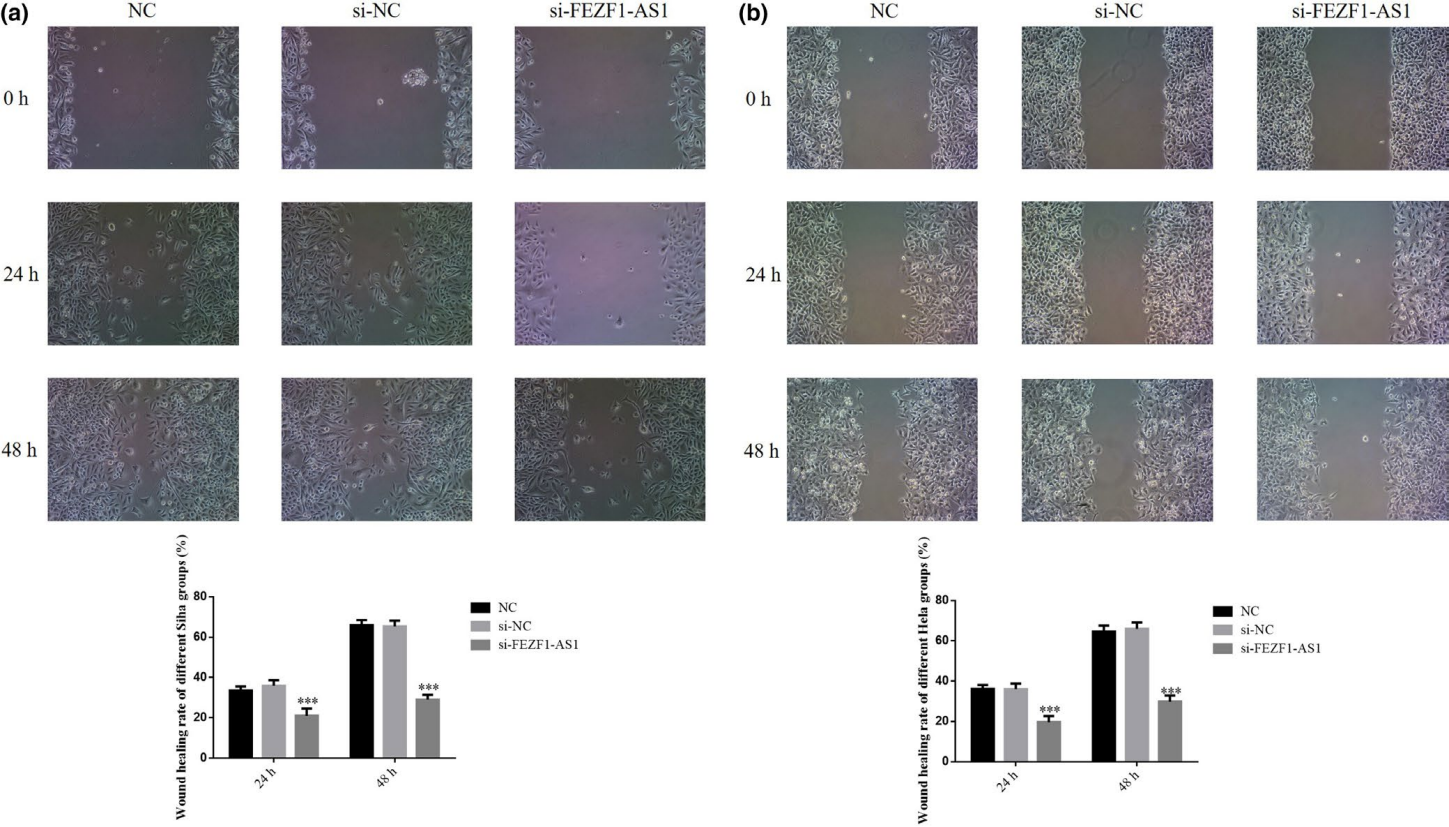
Knockout of FEZF1‐AS1 on migration of CC cells. (a) Wound healing rate of different Siha group. (b) Wound healing rate of different Hela group. ***p <.001, compared with NC

According to the wound healing assay results, the 24‐hr and 48‐hr wound healing rates in the FEZF1‐AS1‐knocked out group were significantly lower than those in the NC group (*p* <.001, Figure 5a&b), while the wound healing rates of SiHa and HeLa cells in the si‐NC group showed no significant difference (*p* >.05).

### 3.6 Influence of the knockout of FEZF1‐AS1 on relevant genes

**FIGURE 6:**
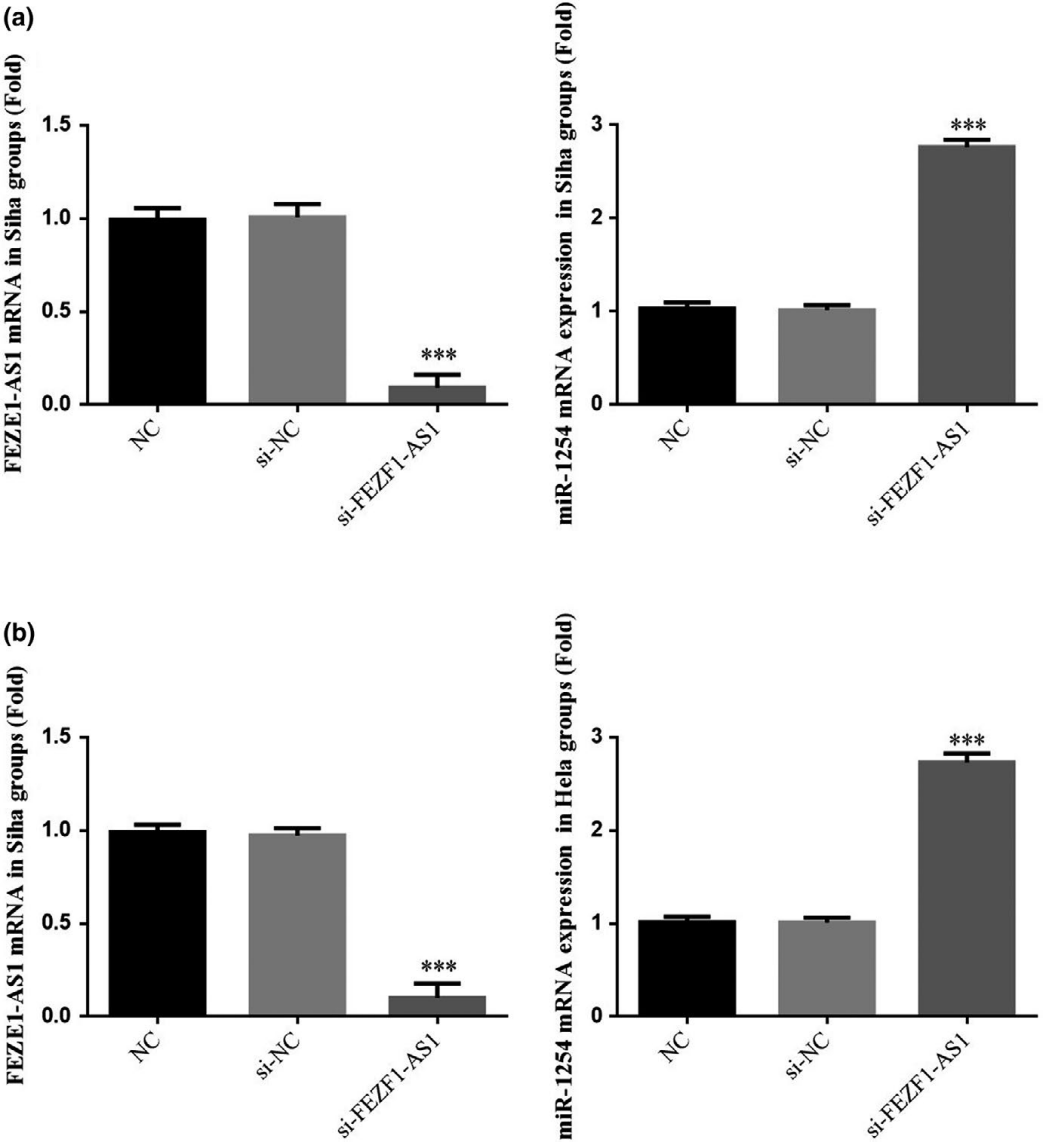
FEZF1‐AS1 and miR‐1254 gene expression by RT‐qPCR assay. (a) FEZF1‐AS1 and miR‐1254 gene expression in Siha group. (b) FEZF1‐AS1 and miR‐1254 gene expression in Hela group. ***p <.001, compared with NC

According to the RT‐qPCR detection, the expression of lncRNA FEZF1‐AS1 gene in the si‐FEZF1‐AS1 group was significantly lower than that in the NC group (*p* <.001, respectively, Figure 6a&b), while the expression of miR‐1254 gene was significantly increased (*p* <.001, respectively, Figure 6a&b). The result indicated that lncRNA FEZF1‐AS1 could negatively regulate miR‐1254 in CC SiHa and HeLa cells.

### 3.7 Influence of the knockout of FEZF1‐AS1 on relevant proteins

**FIGURE 7:**
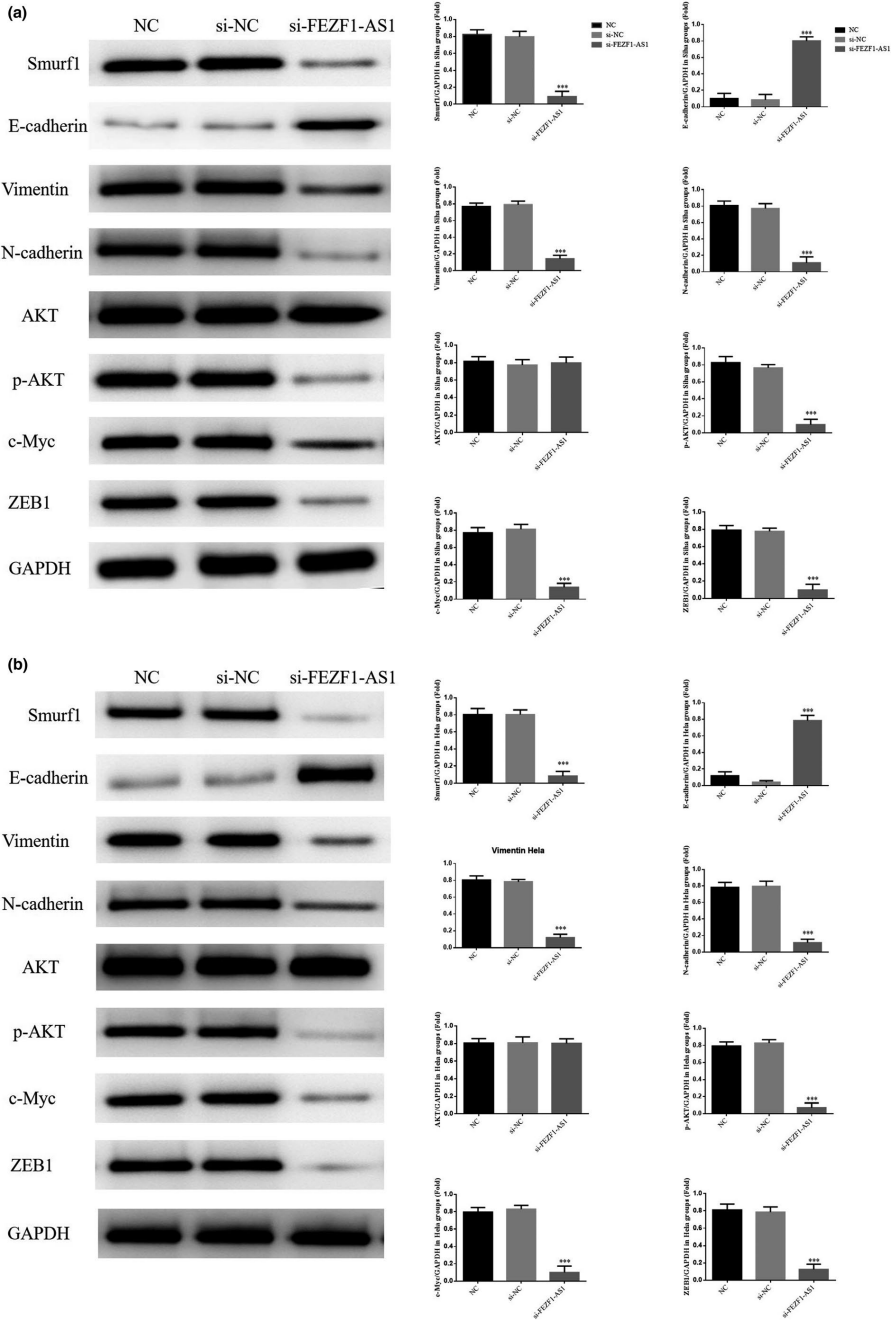
Knockout of FEZF1‐AS1 on relevant proteins. (a) Relative proteins expression in Siha. (b) Relative proteins expression in Hela. ***p <.001, compared with NC group

The results of WB indicated that the expression of Smuf1, Vimentin, N‐cadherin, p‐AKT, c‐Myc, and ZEB1 proteins in the si‐FEZF1‐AS1 group was significantly lower than that in the NC group, while the expression of the E‐cadherin protein increased significantly (*p* <.001, respectively, Figure 7a&b); however, the expression of E‐cadherin, Smuf1, Vimentin, N‐cadherin, p‐AKT, c‐Myc, and ZEB1 proteins in the si‐NC group showed no significant difference (*p* >.05).

### 3.8 Role of miR‐1254 in regulation of CC cell proliferation by si‐FEZF1‐AS1

**FIGURE 8:**
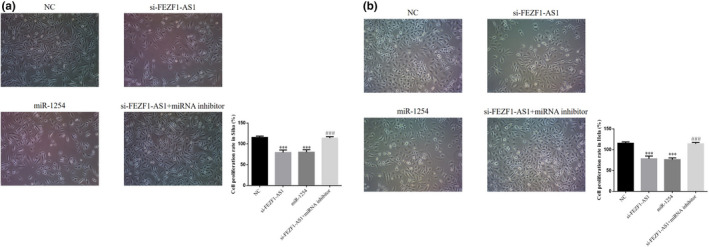
miR‐1254 in regulation of CC cell proliferation by si‐FEZF1‐AS1. (a) Cell proliferation rate in Siha. (b) Cell proliferation rate in Hela. ***p <.001, compared with NC; ###*p* <.001, compared with si‐FEZF1‐AS1

The results of MTT testing showed that the cell proliferation rates in the si‐FEZF1‐AS1 group and the miR‐1254 group were significantly lower than that in the NC group (*p* <.001, respectively, Figure 8a&b); after the cotransfection of si‐FEZF1‐AS1 and miRNA inhibitors, the cell proliferation rate in the si‐FEZF1‐AS1+miRNA inhibitor group was much higher than that in the si‐FEZF1‐AS1 group (*p* <.001, respectively, Figure 8a&b). It can be inferred that the inhibition of CC cell proliferation activity through the knockdown of FEZF1‐AS1 might be realized by upregulating miR‐1254.

### 3.9 Roles of miR‐1254 in regulation of CC cell apoptosis by si‐FEZF1‐AS1

**FIGURE 9:**
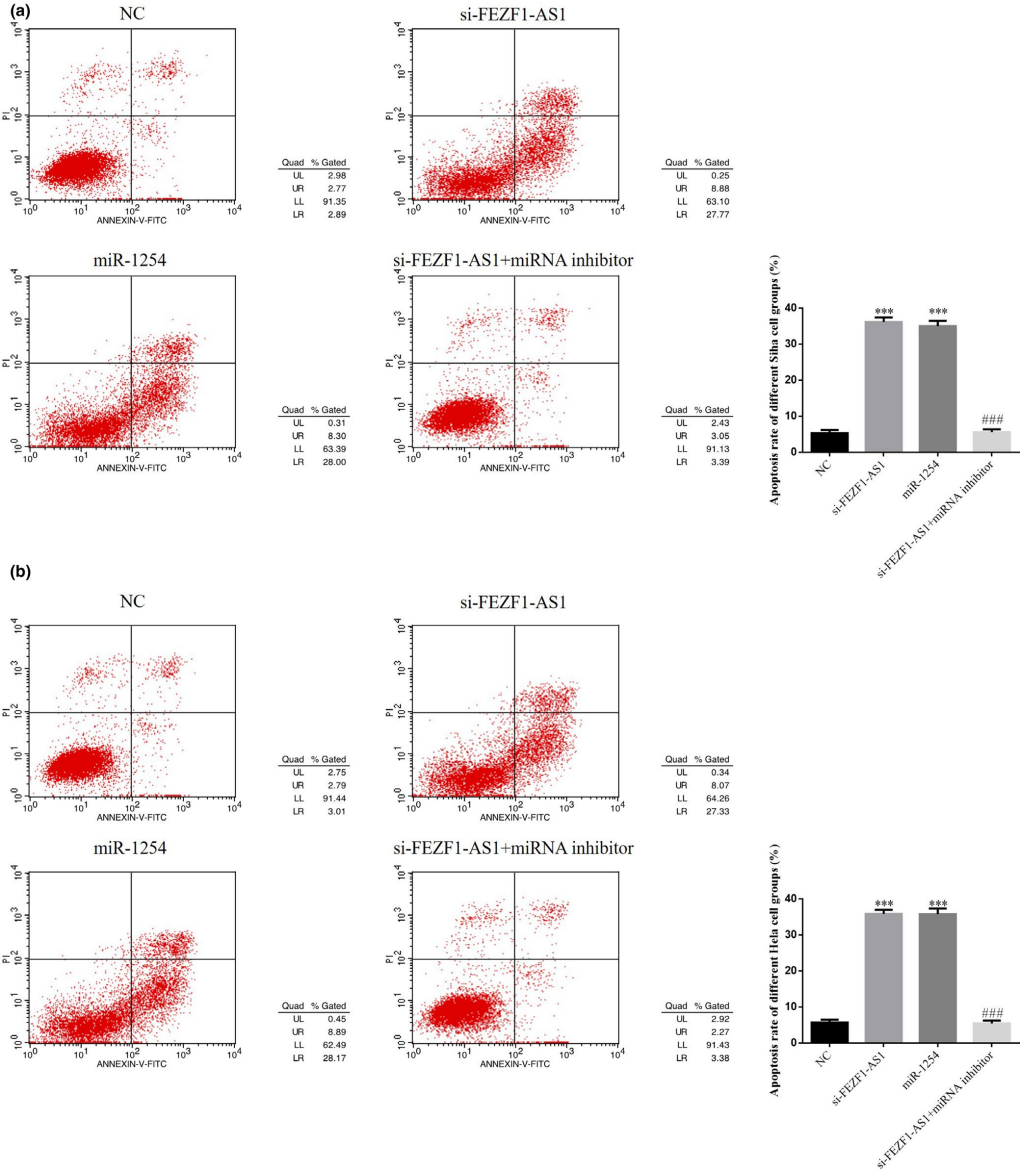
Roles of miR‐1254 in regulation of CC cell apoptosis by si‐FEZF1‐AS1. (a) Apoptosis rate of Siha cell groups. (b) Apoptosis rate of Hela cell groups. ***p <.001, compared with NC; ###*p* <.001, compared with si‐FEZF1‐AS1

The FCM results showed that the cell apoptosis rates in the si‐FEZF1‐AS1 group and the miR‐1254 group were significantly higher than that in the NC group (*p* <.001, respectively, Figure 9a&b); after the cotransfection of si‐FEZF1‐AS1 and miRNA inhibitors, the cell apoptosis rate in the si‐FEZF1‐AS1+miRNA inhibitor group was much lower than that in the si‐FEZF1‐AS1 group (*p* <.001, respectively, Figure 9a&b).

### 3.10 Roles of miR‐1254 in regulation of CC cell invasion by si‐FEZF1‐AS1

**FIGURE 10:**
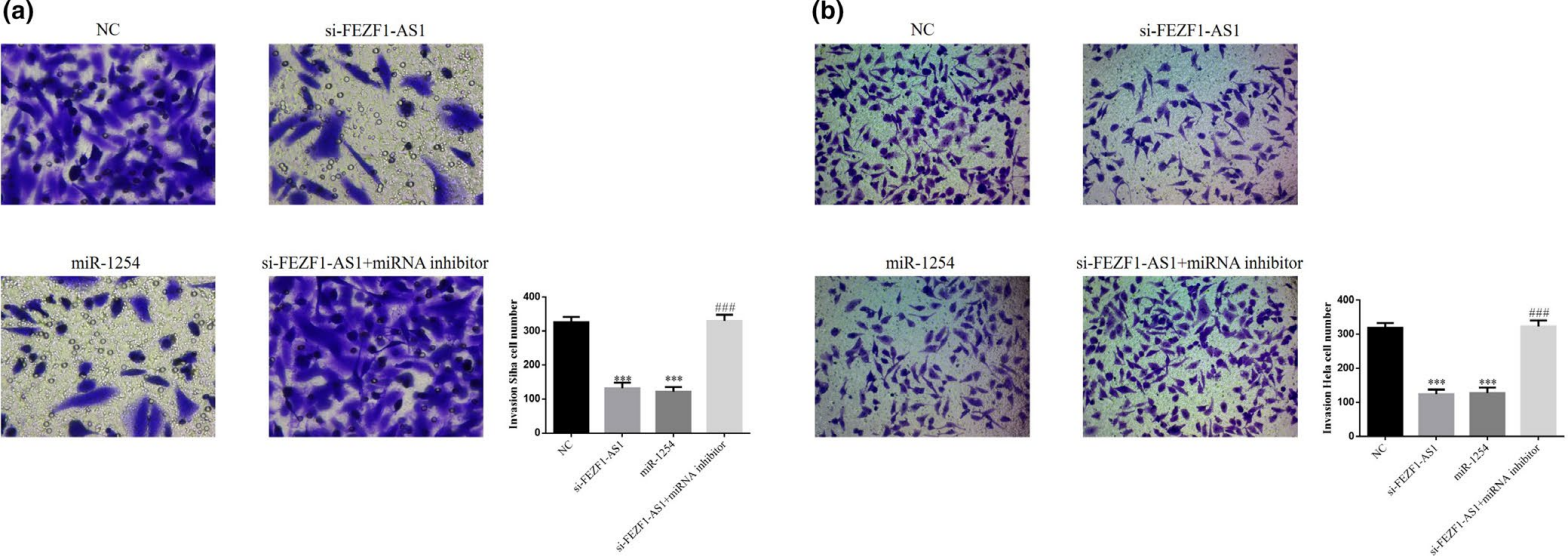
Roles of miR‐1254 in regulation of CC cell invasion by si‐FEZF1‐AS1. (a) Invasion Siha cell number of different groups. (b) Invasion Hela cell number of different groups. ***p <.001, compared with NC; ###*p* <.001, compared with si‐FEZF1‐AS1

According to the Transwell results, the number of invading cells in the si‐FEZF1‐AS1 group and the miR‐1254 group was significantly smaller than that in the NC group (*p* <.001, respectively, Figure 10a&b); after the cotransfection of si‐FEZF1‐AS1 and miRNA inhibitors, the number of invading cells in the si‐FEZF1‐AS1+miRNA inhibitor group was much higher than that in the si‐FEZF1‐AS1 group (*p* <.001, respectively, Figure 10a&b).

### 3.11 Roles of miR‐1254 in regulation of CC cell migration by si‐FEZF1‐AS1

**FIGURE 11:**
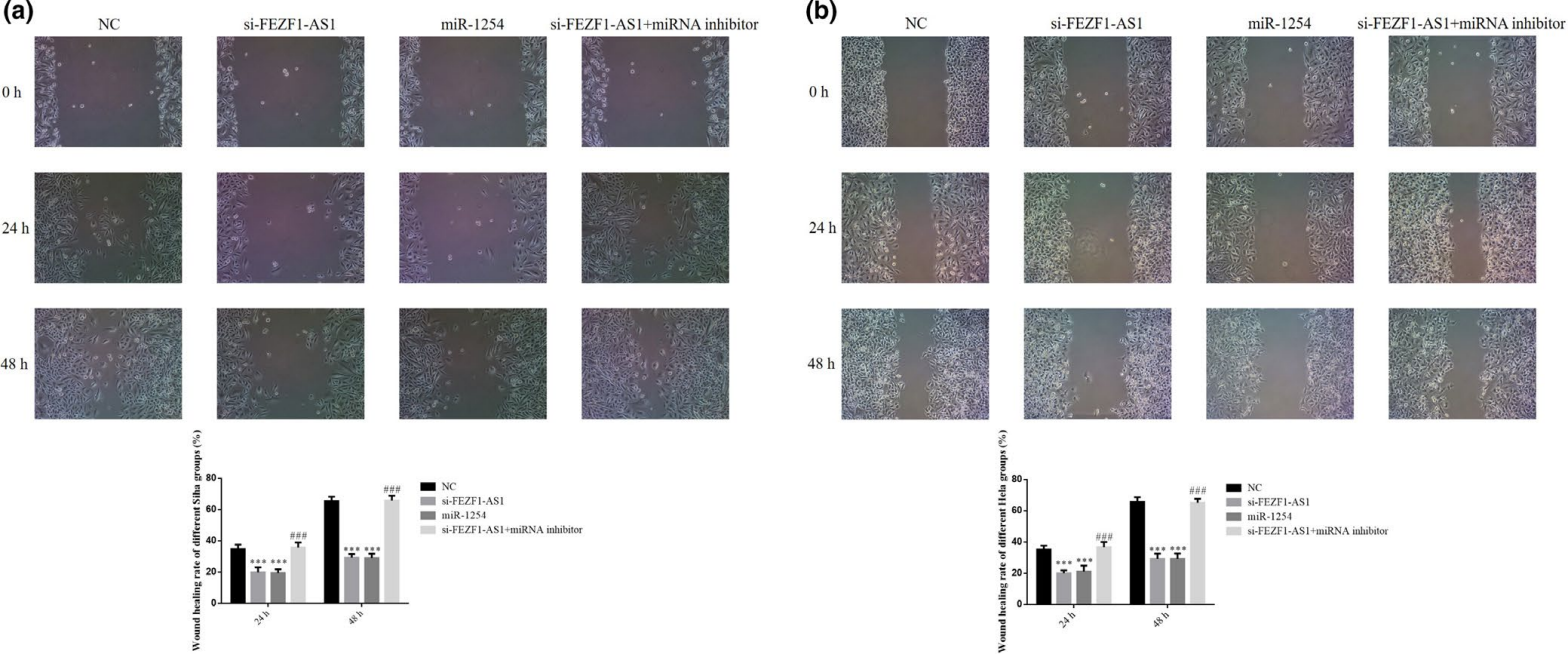
Roles of miR‐1254 in regulation of CC cell migration by si‐FEZF1‐AS1. (a) Wound healing rate of different Siha groups. (b) Wound healing rate of different Hela groups. ***p <.001, compared with NC; ###*p* <.001, compared with si‐FEZF1‐AS1

The results of wound healing showed that the wound healing rates in the si‐FEZF1‐AS1 group and the miR‐1254 group were significantly lower than that in the NC group (*p* <.001, respectively, Figure 11a&b); after the cotransfection of si‐FEZF1‐AS1 and miRNA inhibitors, the wound healing rate in the si‐FEZF1‐AS1+miRNA inhibitor group was much higher than that in the si‐FEZF1‐AS1 group (*p* <.001, respectively, Figure 11a&b).

### 3.12 Expression of FEZF1‐AS1 and miR‐1254 in each group

**FIGURE 12:**
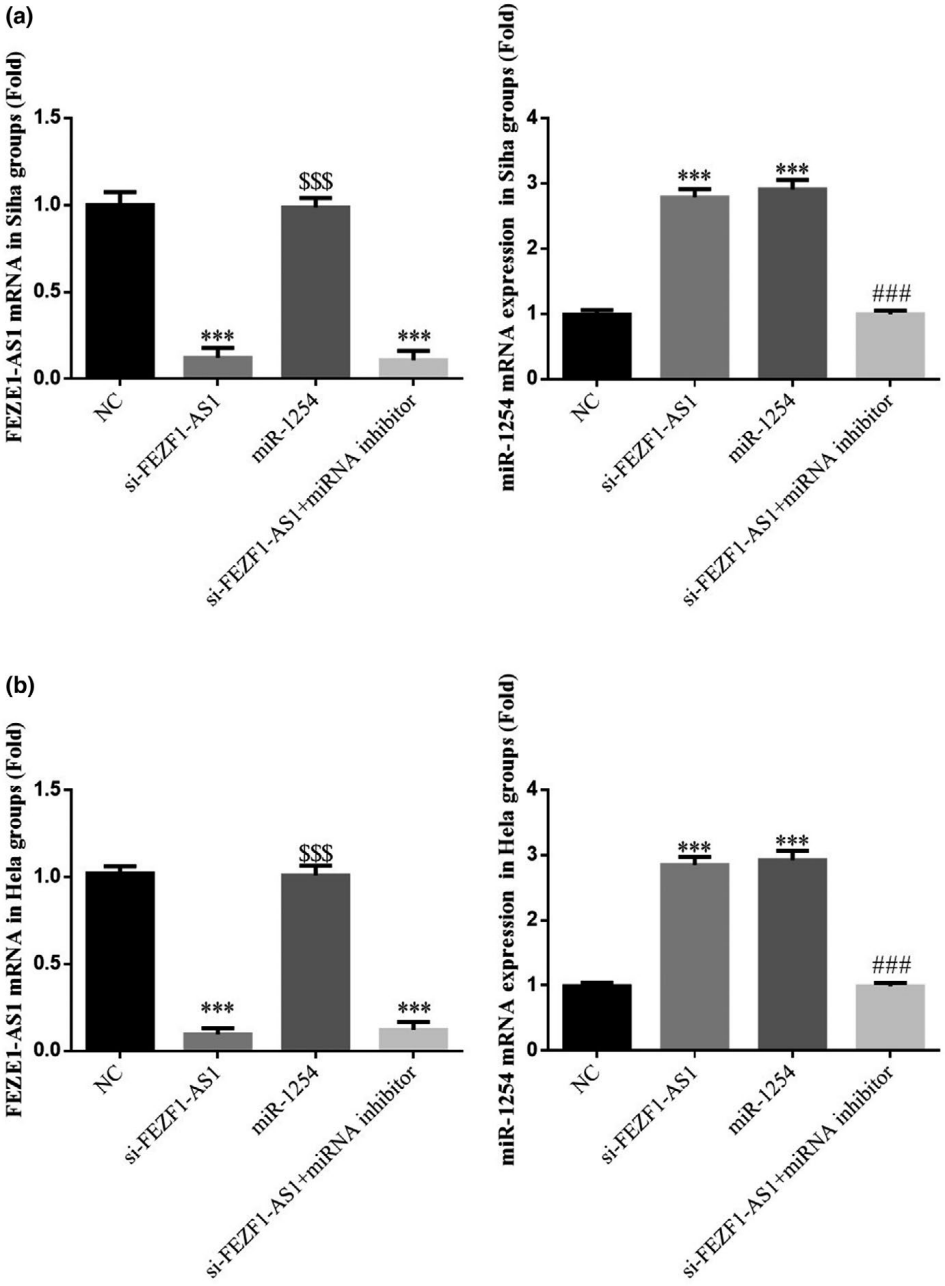
Expression of FEZF1‐AS1 and miR‐1254 in each group. (a) Relative gene expression in Siha cell groups. (b) Relative gene expression in Hela cell groups. ***p <.001, compared with NC; ###*p* <.001, compared with si‐FEZF1‐AS1

The RT‐qPCR detection results indicated that compared with the NC group, the expression of FEZF1‐AS1 gene in the si‐FEZF1‐AS1 group and the si‐FEZF1‐AS1+miRNA inhibitor group was significantly lower (*p* <.001, respectively, Figure 12a&b); and the expression of miR‐1254 gene in the si‐FEZF‐AS1 group and the miR‐1254 group was significantly higher (*p* <.001, respectively, Figure 12a&b); compared with the si‐FEZF1‐AS1 group, the expression of FEZF1‐AS1 gene in the miR‐1254 group was significantly higher (*p* <.001, respectively, Figure 12a&b); the expression of miR‐1254 in the si‐FEZF1‐AS1+miRNA inhibitor group was significantly lower (*p* <.001, respectively, Figure 12a&b).

### 3.13 Relevant proteins by WB assay

**FIGURE 13:**
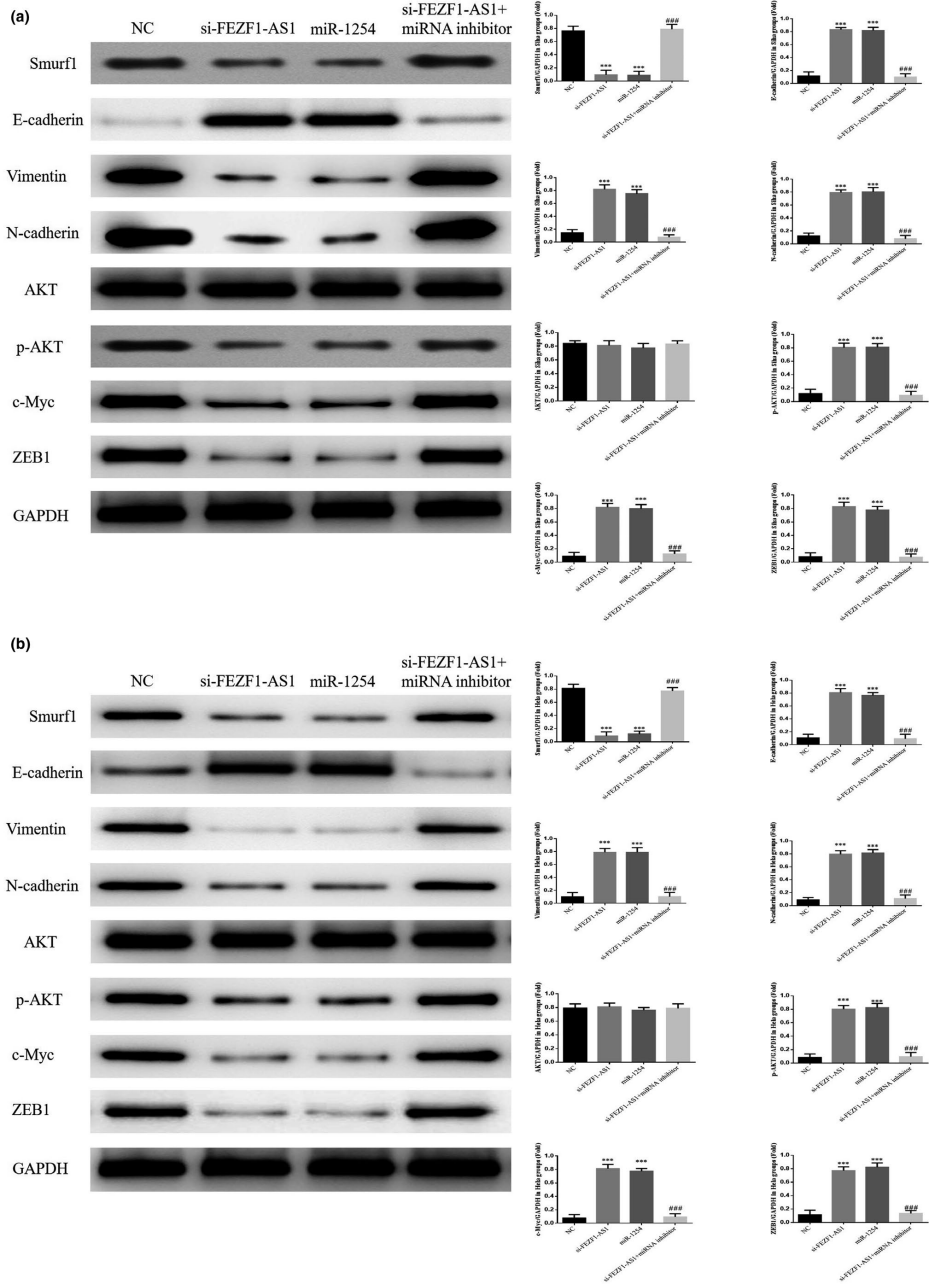
Relevant proteins by WB assay. (a) Relative proteins expression in Siha. (b) Relative protein expression in Hela. ***p <.001, compared with NC; ###: *p* <.001, compared with si‐FEZF1‐AS1

Compared with the NC group, the expression of Smuf1, Vimentin, N‐cadherin, p‐AKT, c‐Myc, and ZEB1 proteins in the si‐FEZF1‐AS1 group and the miR‐1554 group was significantly lower, while the expression of E‐cadherin protein was significantly higher (*p* <.001, respectively, Figure 13a&b); after the cotransfection of si‐FEZF1‐AS1 and miRNA inhibitors, compared with the si‐FEZF1‐AS1 group, the expression of Smuf1, Vimentin, N‐cadherin, p‐AKT, c‐Myc, and ZEB1 proteins in the si‐FEZF1‐AS1+miRNA inhibitor group was significantly higher, while that of E‐cadherin proteins was significantly lower (*p* <.001, respectively, Figure 13a&b).

### 3.14 Targeted regulation of miR‐1254 by FEZF1‐AS1 and targeted regulation of Smurf1 by miR‐1254

**FIGURE 14:**
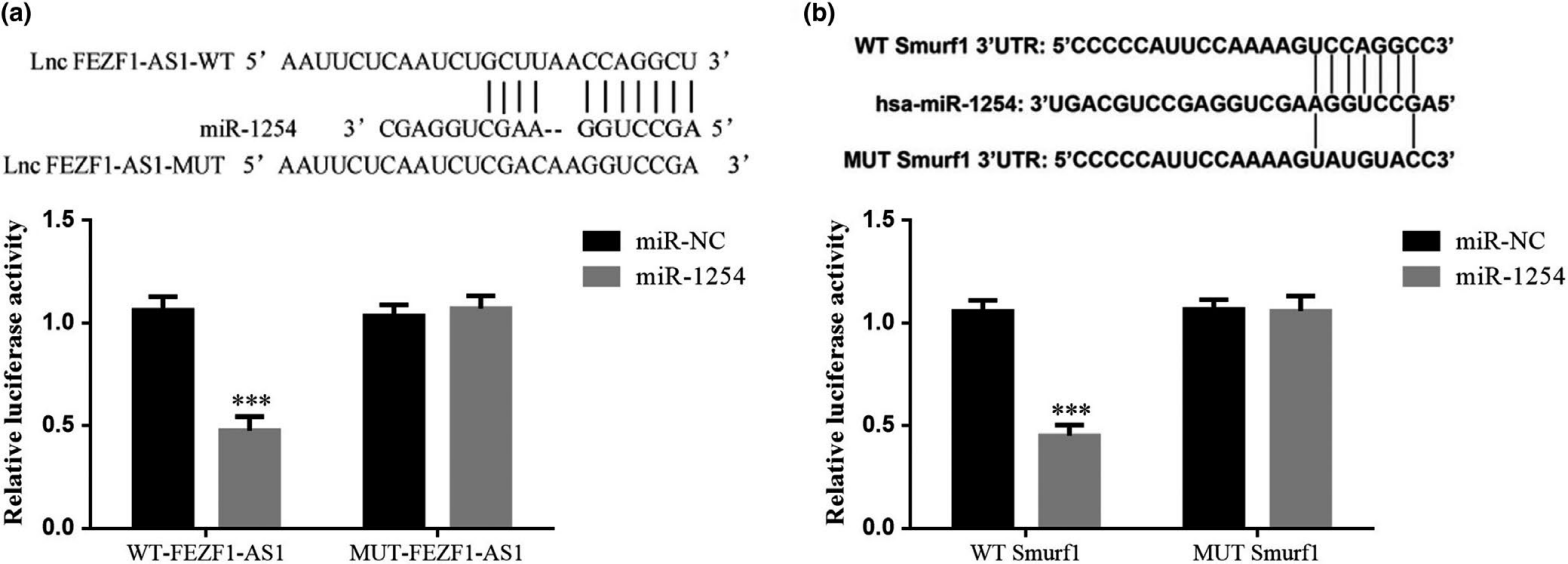
Targeted regulation of miR‐1254 by FEZF1‐AS1 and targeted regulation of Smurf1 by miR‐1254. (a) Correlation between FEZF1‐AS1 and miR‐1254. (b) Correlation between miR‐1254 and Smurf1. ***p <.001, compared with miR‐NC

The dual luciferase reporter gene assay showed that FEZF1‐AS1 could realize targeted regulation of miR‐1254 (Figure 14a); further detection found that miR‐1254 could in turn realize targeted regulation of Smurf1 (Figure 14b).

## 4 DISCUSSION

CC is a malignant tumor of the female reproductive system worldwide and seriously threatens women's health. Research on CC has been gradually refined from the cellular level to the molecular level (Small et al., 2017). As reported by multiple studies, lncRNAs can be closely correlated to tumorigenesis and development by binding to proteins or regulating the expression of miRNAs. Some studies also stressed the carcinogenic features of lncRNAs and lncRNAs’ contributions to the malignant phenotype of CC: lncRNA PCAT6 regulates ZEB1 through the competitive endogenous RNA mechanism to affect the chemotherapy resistance of CC cells (Ma et al., 2020); LINC00511 acts synergistically with the transcription factor RXRA to regulate the expression of PLD1 and promote the autophagy and apoptosis of CC cells (Shi et al., 2020); while lncRNA ANRIL, lncRNA CCHE1, and MEG3 may be closely related to the abnormal proliferation and escape from apoptosis of CC cells (Zhang et al., 2018; Zhu and Han, 2019; Chen et al., 2017). According to a recent study (Bian et al., 2018), the expression of FEZF1‐AS1 in colon cancer tissues and cells was upregulated, and the inhibition of the expression of FEZF1‐AS1 could significantly inhibit the migration, invasion, and proliferation of colon cancer cells and inhibit tumor growth. The results of the present study showed that LncRNA FezF1‐AS1 was overexpressed in CC tissues. The results of clinical analysis indicated that the overexpression of lncRNA FEZF1‐AS1 was closely related to the poor prognosis of patients with CC. The results of cell experiments also confirmed that the knockout of lncRNA FEZF1‐AS1 can effectively inhibit the bioactivity of CC cells. It can be seen that FEZF1‐AS1 can trigger CC, but the molecular mechanism of its roles is still unclear.

In recent years, studies have found that lncRNAs can reduce the expression of target miRNAs by playing the role of miRNA sponges to indirectly regulate genes or mRNA functions. It has been confirmed in studies that FEZF1‐AS1 can regulate the tumor progression by interacting with different miRNAs such as miR‐196a, miR‐107, and miR‐30a in cancers like oral squamous cell carcinoma (OSCC), pancreatic duct adenocarcinoma (PDAC), and breast cancer (Xu et al., 2019; Ye et al., 2018; Zhang et al., 2018). As predicted by target gene databases, miR‐1254 is a target gene of FEZF1‐AS1. Further studies showed that Fezf1‐as1 performs targeted negative regulation on the expression of Mir‐1254. The overexpression of Mir‐1254 can inhibit the proliferation, migration, and invasion of CC cells and the occurrence of EMT and promote cell apoptosis, which is consistent with the antitumor effect of silencing FEZF1‐AS1. Besides, the study found that the interference with miR‐1254 expression can reverse the effects of silencing FEZF1‐AS1 on the proliferation, migration, invasion, EMT occurrence, and apoptosis of CC cells. This indicates that FEZF1‐AS1 may play a pro‐oncogenic role in the progression of liver cancer by targeting miR‐1254.

Previous studies have shown that the abnormally elevated expression of Smurf1 is an important participant in tumorigenesis (Xie et al., 2014). Nevertheless, other studies have shown that Smurf1 can inhibit tumor activity (Li et al., 2017). It was found in this study that after the up‐regulation of miR‐1254 due to the knockout of FEZF1‐AS1, the bioactivity of CC cells was effectively controlled, realizing the targeted inhibition of Smurf1 expression by miR‐1254. Previous studies have shown that the overactive PI3K/AKT signaling pathway is an important factor that leads to the enhancement of abnormal proliferation and metastasis of tumors (Xu et al., 2015). Meanwhile, some reports (Ke et al., 2017) have shown that Smurf1 can regulate the activity of the PI3K/AKT signaling pathway as a regulator. In addition, as the downstream proteins of the PI3K/AKT signaling pathway, c‐Myc and ZEB1 play an important role in regulating the activity (proliferation, invasion, and migration) of tumor cells (Deng et al., 2018; Yuan et al., 2014). According to the results of the study, the reduced expression of c‐Myc and ZEB1 may be an important factor to decrease the bioactivity of CC cells.

The EMT refers to the biological process in which epithelial cells are transformed into specific mesenchymal phenotypic cells by specific procedures (Pastushenko and Blanpain, 2019). During tumorigenesis, the EMT is closely correlated to the invasion, migration, and drug resistance of tumors (Vynckier and Schmidt, 1998). The abnormally expressed E‐cadherin, Vimentin, and N‐cadherin are symbolic proteins of the EMT (Yamashita et al., 2018). In this study, the knockdown of FEZF1‐AS1 could significantly up‐regulate the expression of E‐cadherin and down‐regulate that of Vimentin and N‐cadherin. Further experiments confirmed that the mechanism was closely related to the elevated expression of miR‐1254.

In summary, the results of the study indicated that the abnormal overexpression of FEZF1‐AS1 may be one of the factors that lead to the tumorigenesis and development of CC and that the knockout of FEZF1‐AS1 can effectively inhibit the bioactivity (proliferation, invasion, and migration) of CC cells. The mechanism may be closely correlated to the miR‐1254/Smurf1 axis.
